# Application of advanced technology in traditional Chinese medicine for cancer therapy

**DOI:** 10.3389/fphar.2022.1038063

**Published:** 2022-10-13

**Authors:** Gaofeng Ke, Jia Zhang, Wufeng Gao, Jiayi Chen, Luotong Liu, Simiao Wang, Huan Zhang, Guojun Yan

**Affiliations:** ^1^ Department of Rehabilitation Medicine, The Affiliated Wenling Hospital of Wenzhou Medical University, Wenling, China; ^2^ School of Pharmacy, Jiangsu Provincial Engineering Research Center of Traditional Chinese Medicine External Medication Development and Application, Nanjing University of Chinese Medicine, Nanjing, China; ^3^ School of Life Sciences, Jilin University, Changchun, China

**Keywords:** traditional Chinese medicine, nanotechnology, CRISPR/Cas9, real-time cell-based assay, flow cytometry

## Abstract

Although cancer has seriously threatened people’s health, it is also identified by the World Health Organization as a controllable, treatable and even curable chronic disease. Traditional Chinese medicine (TCM) has been extensively used to treat cancer due to its multiple targets, minimum side effects and potent therapeutic effects, and thus plays an important role in all stages of tumor therapy. With the continuous progress in cancer treatment, the overall efficacy of cancer therapy has been significantly improved, and the survival time of patients has been dramatically prolonged. In recent years, a series of advanced technologies, including nanotechnology, gene editing technology, real-time cell-based assay (RTCA) technology, and flow cytometry analysis technology, have been developed and applied to study TCM for cancer therapy, which efficiently improve the medicinal value of TCM and accelerate the research progress of TCM in cancer therapy. Therefore, the applications of these advanced technologies in TCM for cancer therapy are summarized in this review. We hope this review will provide a good guidance for TCM in cancer therapy.

## Introduction

In recent years, cancer has become the disease with the second highest mortality rate among all diseases in the world, second only to cardiovascular and cerebrovascular diseases (Bhatt et al., 2017). Numerous methods have been established to treat cancer, such as chemotherapy, radiotherapy, surgery, and immunotherapy, etc. (Baskar et al., 2012; Tang et al., 2020). However, all these treatments also suffer from certain limitations. For example, surgery is limited to the minority of cancers, while radiotherapy often causes severe side effects and induces depression and appetite lose in patients (Wu et al., 2013). Chemotherapy is the most common therapeutic method, however, the resistance of tumor cells to the chemotherapy is the main reason that hinders the application of chemotherapy drugs (Tang et al., 2020). Therefore, it is urgent to find alternative drugs to treat tumors.

Nowadays, traditional Chinese medicine (TCM), one of the mainstream forms of alternative drugs, has been widely used to treat cancer due to its multiple targets, minimum side effects and potent therapeutic effects (Cui et al., 2004; Chen et al., 2008; Xie et al., 2016). TCM presents lower toxic effects, higher quality of life, and longer survival rate in cancer therapy (Xiang et al., 2019). Meanwhile, many TCM ingredients extracted from natural plants such as curcumin, resveratrol, berberine, quercetin, tanshinone also show strong antitumor effects including inhibition of the development, proliferation, angiogenesis, and metastasis of human cancer, thus play an important role in all stages of cancer therapy (Hu et al., 2013; Yang et al., 2015; Xu et al., 2016).

Recently, many advanced technologies had been widely applied to investigate TCM for cancer therapy. For example, TCM can be developed into nano-drug delivery system (TCM-NDDS) through nanotechnology (Zhang L et al., 2018; Gao et al., 2019), and the drawbacks of TCM such as poor bioavailability, low solubility, short plasma half-life can be excellent solved, and this will greatly improve the therapeutic value of TCM to treat and prevent cancer (Geng et al., 2022). Meanwhile, gene editing technology also provides a new strategy in TCM for cancer therapy (Rhee et al., 2018). Through gene editing technology, the molecular mechanisms underlying TCM for cancer therapy can be studied in detail, animal models can be established by this novel method, and all these will significantly reduce the difficulties in tumor therapy research (Concha-Benavente and Ferris, 2017; Shen, et al., 2021). Moreover, a series of physiological states such as cell viability, migration, and growth changes can be reflected in a real time way by real-time cell-based assay (RTCA) technology with the advantages of high accuracy and repeatability (Wang T. X, et al., 2013; Yan et al., 2018). In addition, flow cytometry analysis technology, one of the qualitative and quantitative analysis of advanced cell technology, is applied to quickly determine the biological properties of cells or biological particle under a fast linear flow state in TCM study due to its high sensitivity, fast analysis, high accuracy, wide measurement parameters and high throughput (Adan et al., 2017; McKinnon, 2018; Feng and Zhao, 2021). These advanced technologies have been extensively applied in the researches of lymphoma, cervical cancer and other solid tumors, thus promoting the development of TCM (Concha-Benavente and Ferris, 2017; Shi M. et al., 2021).

**FIGURE 1:**
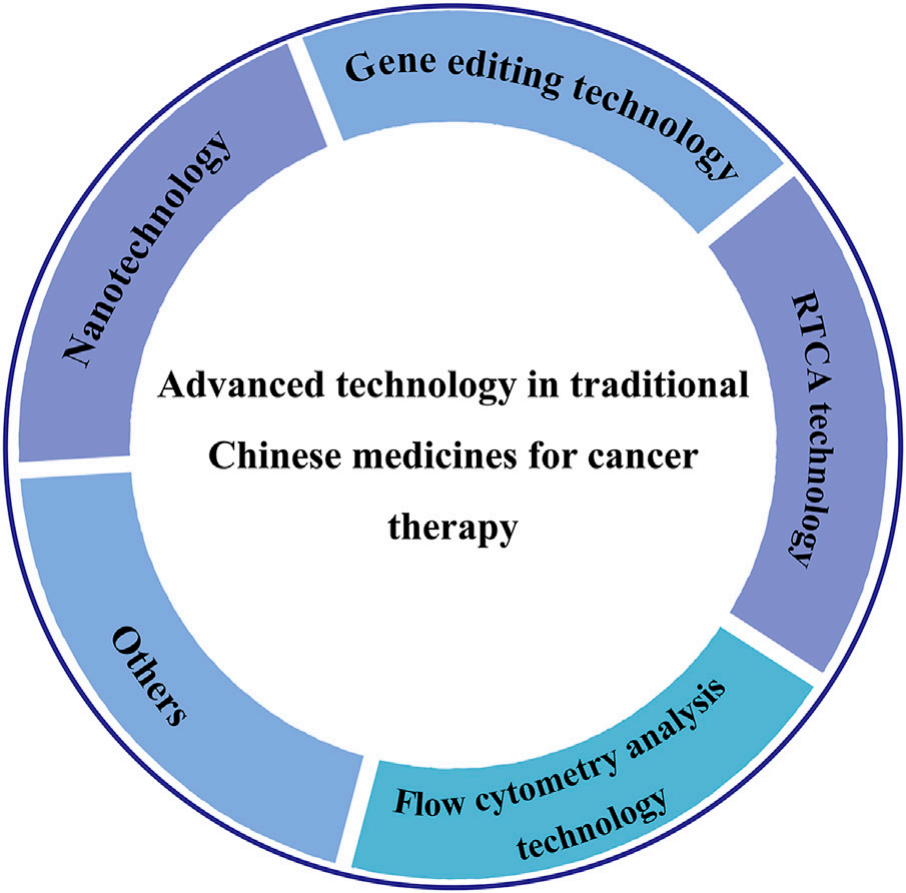
The main topics of this review. The main topics include the application of advanced technologies such as nanotechnology, gene editing technology, RTCA technology, flow cytometry analysis technology in TCM for cancer therapy.

As is known to all, advanced technology makes great contributions in TCM for cancer therapy, and thus promoting the rapid development of TCM, and the shortcomings of TCM in actual application are also maken up by interdisciplinary research (Zheng et al., 2022). Therefore, in order to efficiently enhance the therapeutic value of TCM and accelerate the research progress of TCM in cancer therapy, application of advanced technology (e.g., nanotechnology, gene editing technology, RTCA technology, flow cytometry analysis technology, etc.) in TCM for cancer therapy were summarized, and the main areas of this review is summarized in Figure 1.

## The application of advanced technology

Many advanced technologies including nanotechnology, gene editing technology, RTCA technology, flow cytometry analysis technology had been extensively used to investigate the effect of TCM for cancer therapy, as shown in Table 1. In this section, we summarize the research development of these technologies in recent years.

**TABLE 1:** The application of advanced technology in tumor treatment research.

TCM	Cancer	Advanced technology	Application	Ref
Oleanolic acid	Human ovarian cancer	Nanotechnology	Improve the treatment efficiency of the hydrophobic	Zhang L et al. (2018)
			Chinese medicine component	
Isoimperatorin	Lymphoma	Nanotechnology	Improve the therapeutic effect of lymphoma	Zhao et al. (2021)
Quercetin	Breast cancer	Nanotechnology	Improve tumor targeting and radiosensitivity	Huang et al. (2020)
Quercetin	Breast cancer	Nanotechnology	Improve antitumor efficiency and specificity	Zhang Q et al. (2018)
Celastrol	Melanoma	Nanotechnology	Enhance anticancer efficacy of Celastrol	Geng et al. (2022)
10-Hydroxycam-ptothecin	Glioma	Nanotechnology	Enhance anticancer efficacy of 10-Hydroxycamptothecin	Gao et al. (2019)
Bufalin	Insensitive glioma	CRISPR/Cas9 Technology	Determine the roles of the proteins in apoptosis and necroptosis	Hu et al. (2013)
Loureirin B	Autoimmune diseases	CRISPR/Cas9 Technology	Edit K(V)1.3 coding gene KCNA3 and successfully generated a K(V)1.3 knockout (KO) cell model	Shi S. J. et al. (2021)
Isobavachalcone	Acute myeloid leukemia	CRISPR/Cas9 Technology	Demonstrate that CRISPR/Cas9-mediated knockout of dihydroorotate dehydrogenase leads to apoptosis and normal differentiation of acute myeloid leukemia cells	Wu et al. (2018)
Chrysin	Gastric cancer	CRISPR/Cas9 Technology	Knock out the TET1 gene	Zhong et al. (2020)
Galangin	Hepatocellular carcinoma	CRISPR/Cas9 Technology	Analyze loss expression of H19 *in vivo*	Zhong et al. (2020)
Dioscin	Colorectal cancer	CRISPR/Cas9 Technology	Knockout of the F-box protein S-phase kinase-associated protein 2 (Skp2)	Zhou et al. (2020)
Chelidonine hydrochloride	Cervical cancer	RTCA technology	Analyze the inhibitory ability of chelidonine hydrochloride on cervical cancer cells	Yang et al. (2020)
Coumarin compound isoimperatorin	Nasopharyngeal carcinoma	RTCA technology	Monitor the proliferation of nasopharyngeal carcinoma CNE2 cells in real time	Liu et al. (2020)
Curcumin	Gastric cancer	RTCA technology	Evaluate the effect of curcumin micelles on gastric cancer cell growth	Wang S. Y et al. (2013)
Oleandrin	Breast cancer	RTCA technology	Investigate the anti-breast cancer effect of oleandrin	Li et al. (2020)
Dihydroartemisinin	Gastric cancer	Flow cytometry analysis technology	Evaluate the gastric cancer cell proliferation	Fan et al. (2020)
Curcumin	Renal carcinoma	Flow cytometry analysis technology	Evaluate the apoptosis of renal carcinoma cells	Gong et al. (2021)
Puerarin	Hepatoma	Flow cytometry analysis technology	Detect the effects of different concentrations puerarin on the survival and apoptosis of SMMC-7721cells	Zhang et al. (2017)
Osthole	Human tongue squamous cell carcinoma	Flow cytometry analysis technology	Detect the apoptosis of human tongue squamous cell	Sun and Liu, (2021)
Trichosanthin	Cervical cancer	Flow cytometry analysis technology	Investigate the role of trichosanthin in cervical cancer	Zhu et al. (2021)
Dongxia pill	Nasopharyngeal carcinoma	Flow cytometry analysis technology	Investigate the effect of Dongxia Pill on tumor cell	Huang et al. (1999)

### Nanotechnology

As is well known, the therapeutic effects of TCM are mainly attributable to the active ingredients inside (Li et al., 2018). According to their differences in chemical structures, the ingredients in TCM can be classified into terpenoids, alkaloids, sesquiterpenes, polysaccharides, glycosides, saponins, etc. (Puglia et al., 2017). However, the physicochemical properties of some active ingredients such as poor solubility, low bioavailability, short plasma half-life may limit their application (Gao et al., 2019). In order to solve these drawbacks, nanotechnology was introduced into TCM in recently years (Zhang Q et al., 2018; Gao et al., 2019; Bayda et al., 2020). Nanotechnology is a science that involves detection, design, fabrication, and application of certain chemicals and materials on the level of nanoscale. TCM can be carefully developed into TCM-NDDS through nanotechnology, which will greatly enhance the therapeutic value of TCM to treat and even prevent cancer (Ma et al., 2019).

**FIGURE 2:**
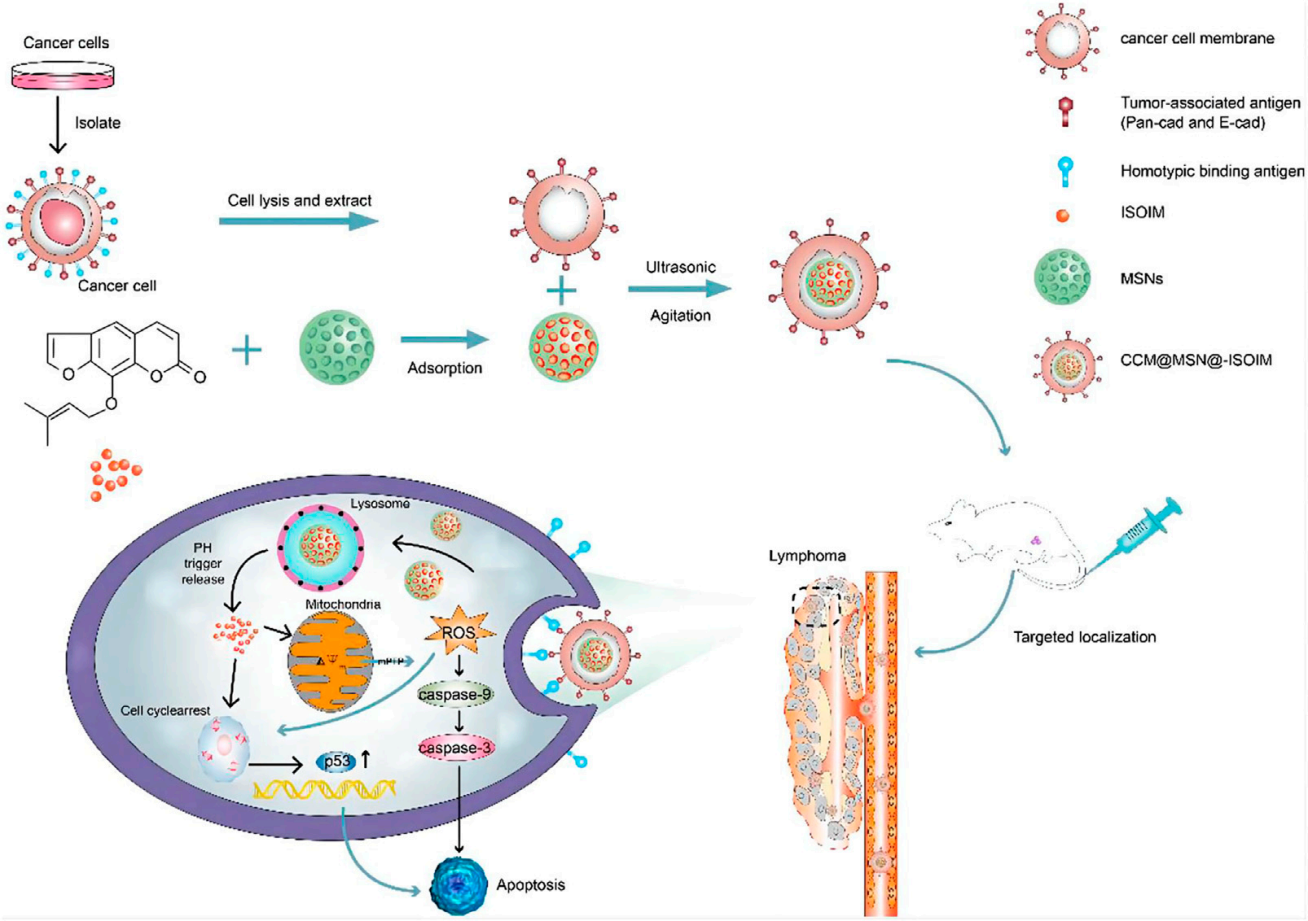
The scheme diagram of CCM@MSNs-ISOIM construction and its targeted therapeutic mechanisms in lymphoma (Zhao et al., 2021). In this work, a nano-platform based on MSNs which camouflaged by the cancer cell membrane was constructed. The nano-platform has the characteristics of anti-phagocytosis, high drug-loading capacity, low pH sensitivity, good biocompatibility and active targeting, promoting mitochondrial-mediated apoptosis.

Various TCM-NDDS (e.g., liposomes, nanoparticles, micelles, polymer nanoparticles, inorganic nanomaterials) have been meticulously designed to improve the therapeutic value of TCM by nanotechnology, as summarized in Table 1. For example, Zhang et al. (Zhang L et al., 2018) designed and synthesized TCM-NDDS which used hollow nanocages MOF-5 as drug carrier to load oleanolic acid (MOF@OA). Its drug-loading capacity and cytotoxicity were evaluated, and results showed that MOF improved the drug-loading capacity of OA and the kinetic and therapeutic efficiency of OA. Similarly, Zhao et al. (Zhao et al., 2021) constructed a nanocarrier using the mesoporous silica nanoparticles (MSNs) which was further modified by the cancer cell plasma membrane. After modification, TCM isoimperatorin (ISOIM) was loaded inside the empty nanocarrierinside (CCM@MSNs-ISOIM). Their antitumor effects, anti-lymphoma mechanism, and the biosafety *in vivo* were characterized (the prepared scheme and targeted therapeutic mechanisms in lymphoma of CCM@MSNs-ISOIM were presented in Figure 2). This nano-platform presented high drug-loading capacity, good biocompatibility and excellent tumor targeting. Meanwhile, it also showed the characteristics of anti-phagocytosis and low pH sensitivity.

Additionally, in order to overcome the critical drawbacks of celastrol (e.g., low water-solubility, short plasma half-life, and notable systemic toxicity) and improve its antitumor efficacy, Geng et al. (Geng et al., 2022) designed a novel polymer structure, poly (2-(N-oxide-N, N-dimethylamino)ethylmethacrylate)-block-poly (2-hydroxy- ethyl methacrylate) (OPDMA-HEMA), and used it for drug delivery (OPDMA-Cela). The OPDMA-Cela resulted in potent immunogenic cell death and decreased the PD-L1 protein level. Moreover, compared with free celastrol, OPDMA-Cela showed a higher anticancer efficacy, minimum systemic toxicity with enhanced solubility in water. Similarly, Gao et al. (Gao et al., 2019) prepared menthol-modified casein nanocarrier loaded with 10-hydroxycamptothecin (HCPT-M-CA-NPs) for glioma targeting therapy. The results showed that the drug accumulation in the tumor site was greatly improved by HCPT-M-CA-NPs, and the median survival time of intracranial glioma-bearing mice was significantly extended.

In summary, nanotechnology, as an emerging technology in recent years, has received extensive attention from researchers. TCM designed and developed into nano-drug delivery system through nanotechnology can efficiently solved the drawbacks of TCM including poor solubility, low bioavailability, short half-life, thus enhancing the therapeutic use of TCM to treat and prevent cancer.

### Gene editing technology

As an emerging technology, gene editing technology is also extensively applied in cancer therapy in TCM. Gene editing technology is a technology that artificially inserts, deletes and replaces gene sites containing genetic information in the nucleotide sequence (Rhee et al., 2018). It is mainly composed of zinc finger nucleases (ZFNs) technology, transcription activator-like effector nucleases (TALENs) technology and clustered regularly interspaced short palindromic repeats/CRISPR-associated protein (CRISPR/Cas) technology (Puchta et al., 1993; Rouet et al., 1994). At present, due to the complex experimental process and high off-target effect, ZFNs and TALENs technologies have been gradually replaced by CRISPR-Cas technology. Therefore, we will focus our discussion on CRISPR/Cas technology in this review (Adli, 2018).

**FIGURE 3:**
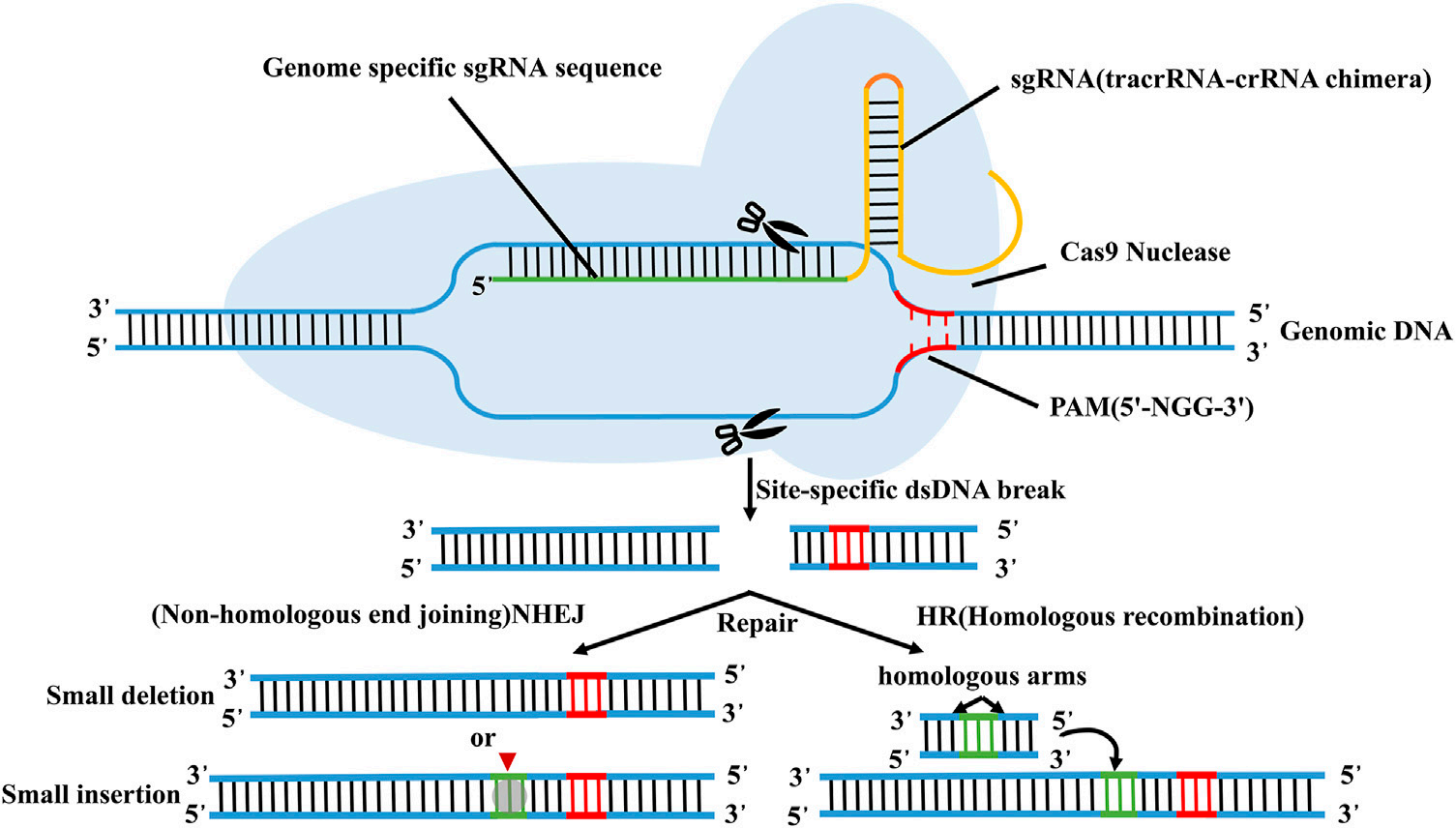
Schematic diagram of CRISPR/Cas9 technology. According to the dimer structure of crRNA and tracrRNA, a new single-stranded chimeric sgRNA (single guide RNA) can be designed to specifically identify the target gene sequence, and then further combined with Cas9 protein to form a targeted cutting complex which specifically cut the target DNA and break the double strand of DNA.

CRISPR/Cas9 technology, as the major type of CRISPR-Cas technology, has been extensively applied in cancer research because of its simple design, high efficiency, low cost and simultaneous multi-point editing (Gesner et al., 2011; Sapranauskas et al., 2011; Sashital et al., 2011). CRISPR/Cas9 system is mainly composed of trans-activated RNA gene expression CRISPR RNA (trans-activating crRNA, tracrRNA), Cas9 protein-coding gene with endonuclease, and specific targeting CRISPR RNA (crRNA) (Jinek et al., 2012; Wen et al., 2015). Its principle is very simple, as shown in Figure 3 (El-Mounadi et al., 2020). According to the dimer structure of crRNA and tracrRNA, a new single-stranded chimeric sgRNA (single guide RNA) can be designed to specifically identify the target gene sequence, and then further combined with Cas9 protein to form a targeted cutting complex which specifically cut the target DNA and break the double strand of DNA (Bhaya et al., 2011; Jinek, Chylinski et al., 2012; Jackson et al., 2014; Qu et al., 2015). In theory, CRISPR/Cas9 technology can achieve editing of any genomic site by synthesizing sgRNA that targets to various genomic sites (Concha-Benavente and Ferris, 2017; Shi S. J. et al., 2021).

CRISPR/Cas9 technology provides a new strategy in TCM for cancer therapy, thus promoting the research progress of tumor therapy in recent years (Maus et al., 2014; Corrales et al., 2017; Ren and Zhao, 2017). It can significantly reduce the difficulties of tumor therapy research and has been extensively applied to the research of lymphoma, cervical cancer and other solid tumors (Concha-Benavente and Ferris, 2017; Shi M. et al., 2021). In order to investigate the roles of proteins in apoptosis and necroptosis, Hu et al. (Hu et al., 2013) knocked out the related gene by CRISPR/Cas9 technology. The results revealed that necrosomes, which is composed of receptor-interacting protein kinase 1, receptor-interacting protein 3 and mixed lineage kinase domain-like protein, underwent necroptosis when caspase-8 function was lost. They further investigated the cytotoxicity of bufalin (a component of TCM) in glioma cell with the gene knockout by CRISPR/Cas9. The results indicated that glioma cells with the knockout of caspase-8 were more sensitive to bufalin than those without caspase-8, and bufalin may be a potential drug for glioma therapy. Shi et al. (Shi S. J. et al., 2021) edited KV1.3 coding gene KCNA3 through this technology, and a KV1.3 knockout (KO) cell model was successfully generated. The results showed that Loureirin B, a component extracted from Resina Draconis, could inhibit Ca^2+^ influx and IL-2 secretion in Jurkat T cells through the inhibition of KV1.3 and STIM1/Orail targets. In addition, in order to explore new targeting, Wu et al. (Wu et al., 2018) knocked-down of dihydroorotate dehydrogenase by CRISPR/Cas9 technology, which resulted in apoptosis and normal differentiation of acute myeloid leukemia cells. This study indicated that dihydroorotate dehydrogenase may be a potential therapeutic target in acute myeloid leukemia. Moreover, they further verified that isobavachalcone, which is derived from psoralea corylifolia, could act as an advanced dihydroorotate dehydrogenase inhibitor to trigger apoptosis and differentiation of acute myeloid leukemia cells, and offered a useful therapeutic target for acute myeloid leukemia treatment. In addition, Zhong et al. (Zhong et al., 2020) used CRISPR/Cas9 technology to knockout ten-eleven translocation 1 (TET1) gene and investigated the effects of chrysin, a natural flavonoid commonly found in honey, with the protein expression of TET in gastric cancer (GC) cells. The results indicated that chrysin was effective against tumors by regulating TET1 expression in GC. Zhong et al. (Zhong et al., 2020) showed that knockdown of H19 and miR675 by CRISPR/Cas9 technology could induced p53 expression and ultimately promoted cell apoptosis in MHCC97H cells. Moreover, Galangin could also promote cell apoptosis by inducing H19 and miR675 expression in MHCC97H cells, presenting a crucial role in hepatocarcinogenesis. Zhou et al. (Zhou et al., 2020) found that knockout of the F-box protein S-phase kinase-associated protein 2 (Skp2) by CRISPR/Cas9 technology could inhibit Hexokinase 2 (HK2) and glycolysis, and affected colorectal cancer cell growth rate *in vitro* and *in vivo*. Dioscin, which is a natural steroid saponin derived from a variety of plants, could reduce the Skp2 protein expression by reducing the stability of Skp2, promoting Skp2 ubiquitination, inhibiting Skp2 expression and thus delaying tumor growth *in vivo*, and all these suggested that enhancement of ubiquitination-dependent Skp2 turnover had great potential for cancer therapy. In summary, all these samples suggest the great potential of CRISPR/Cas9 technology in the TCM study.

For CRISPR/Cas9 technology, it mainly relies on a sgRNA-Cas9 complex, thereby avoiding time-consuming and labor-intensive protein construction process (Ding et al., 2013; Shah et al., 2013; Gupta and Musunuuru, 2014). Meanwhile, it also presents the advantages of simple operation, high gene editing efficiency and good versatility (Cong et al., 2013; Chen et al., 2015). At present, CRISPR/Cas9 technology as an advanced technology was extensively applied in TCM for cancer therapy, promoting its research progress. Though this technology has the aforementioned advantages, CRISPR/Cas9 technology also has some limitations. For example, this technology has a high off-target efficiency, which may lead to the binding of sgRNA to other sites. Meanwhile, the off-target site is generally difficult to find, and thus the whole-genome sequence should be evaluated to determine the off-target site. In addition, the T7 promoter contained in sgRNA synthesized *in vitro* is limited to the transcribed target gene sequence, which limits the selection of target sites to a certain extent (Qiu, 2016; Lei et al., 2020).

### Real-time cell-based assay technology

As a new cell detection technology, RTCA technology is a transient cell sensing continuous recording system based on the principle of resistance and impedance (Wang T. X et al., 2013). RTCA system integrates the microelectronic cell sensor chip into the bottom of the cell detection plate which is suitable for cell attachment and growth. And then, a real-time, dynamic and quantitative cell impedance detection sensing system that tracks changes in cell morphology, proliferation and differentiation is constructed (Stefanowicz-Hajduk et al., 2016). The biological state and changes of cells, and cell dynamics in real time without labeling and damage are monitored by RTCA (Turker sener et al., 2017).

**FIGURE 4:**
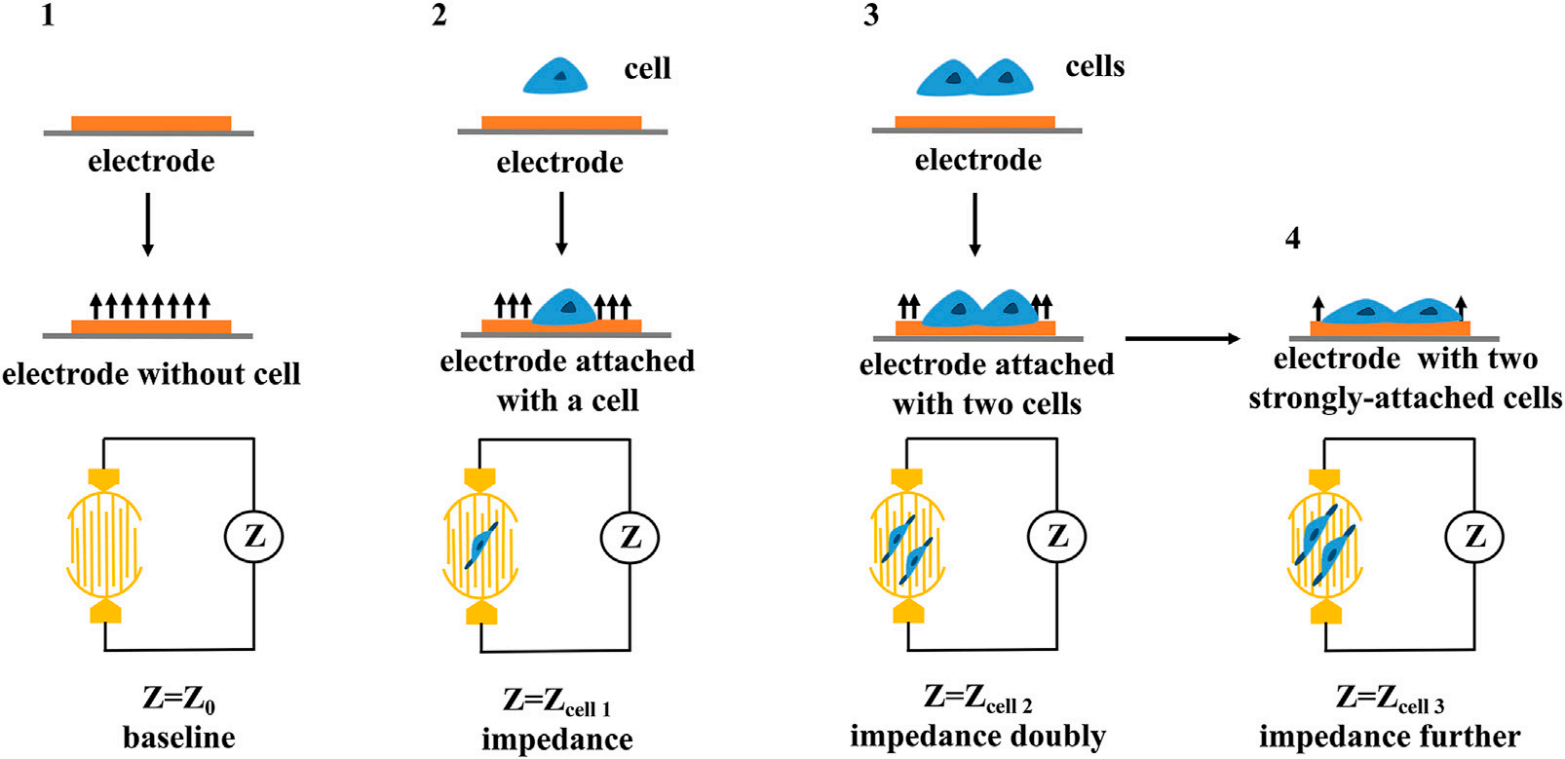
The principle of cellular impedance detection. The cell biological states including quantity, mass, size, attachment state can be reflected by the changes in impedance.

RTCA is mainly composed of biosensor board, impedance measurement unit, impedance conversion unit, real-time analysis and data processing unit (Wang S. Y et al., 2013). The electrical impedance measured by the biosensor board can detect and evaluate the growth state, morphological changes, death number and adhesion degree of cells in real time (Burmakina et al., 2018). The electrode impedance measured by the impedance measurement unit is mainly determined by the ionic environment on the electrode surface and the bulk solution (Meindl et al., 2013), and thus, changes in impedance could reflect changes in cell biological states (e.g., quantity, mass, size, attachment state) (as shown in Figure 4). The impedance conversion unit is mainly composed of analog-to-digital converter (ADC). When the cell condition changes, ADC can record the analog electrical signal in real time manner and automatically convert it into digital signal, which can reflect a series of biological conditions such as cell viability, migration, and growth changes (Wang T. X et al., 2013).

Compared with traditional methods, RTCA has many advantages such as less cell consumption, high throughput, rapid and simple operation (Meindl et al., 2013; Kho et al., 2015). It can be used to verify the function and growth rule of different types of cells and the mechanism of different drugs on tumor cells (Kho et al., 2015), thus RTCA was widely applied to various tumor therapy. Yang et al. (Yang et al., 2020) analyzed the inhibitory ability of different concentrations of chelidonine hydrochloride on cervical cancer cells by combining MTT method, xCELLigence RTCA S16 and colony formation assay. The results showed that the inhibitory effect of chelidonine hydrochloride on cervical cancer cells was dose-dependent, suggesting chelidonine hydrochloride had obvious antitumor effect. In addition, Liu et al. (Liu et al., 2020) applied RTCA technology to monitor the proliferation of nasopharyngeal carcinoma CNE2 cells in a real time, and found that different concentrations of coumarin compound isoimperatorin had significant inhibitory effect on nasopharyngeal carcinoma CNE2 cells. Calibasi-Kocal et al. (Calibasi-Kocal et al., 2019) found that curcumin had anti-proliferation activity against both HCT-116 and metastatic colorectal cancer cells by xCELLigence RTCA DP analysis technique. Lin et al. (Lin et al., 2020) evaluated the effect of curcumin micelles on GC cell growth by combing MTS cell proliferation assay, flow cytometry, RTCA and xenotransplantation. The results showed that curcumin micelles could significantly inhibit cell proliferation, colony formation and apoptosis of GC. Curcumin micelles could also significantly inhibit tumor growth *in vivo*, suggesting that curcumin could be used in the treatment of GC therapy. Li et al. (Li et al., 2020) investigated the anti-breast cancer effect (e.g., MCF10A, MCF7, SK-BR-3 and MDA-MB-231) of oleandrin derived from oleander leaves by RTCA technology. The results revealed that oleandrin exhibited no detectable effect on MCF10A, and had a certain inhibiting effect on other breast cancer cell. Similarly, Yang et al. (Yang et al., 2014) detected the inhibitory effect of salvia miltiorrhiza and ginseng on MCF-7 by RTCA technology. The salvia miltiorrhiza and ginseng presented a continuous inhibitory effect on MCF-7. Meanwhile, the combination of salvia miltiorrhiza and ginseng had a certain selectivity for breast cancer therapy.

RTCA technology overcomes the limitations of the traditional methods such as long time, large interference, and large damage to cells. Moreover, most cells in micropores can be detected in a short time and the detection data has higher accuracy and repeatability (Otero-Gonzalez et al., 2012; Yan et al., 2018). However, RTCA can only indirectly monitor the activity of adherent cells based on cellular impedance, suspended cells require some auxiliary methods (Martinez-Serra et al., 2014). In addition, special microplates are required when the RTCA instruments are used in different experiments, therefore the cost will be increased.

### Flow cytometry analysis technology

**FIGURE 5:**
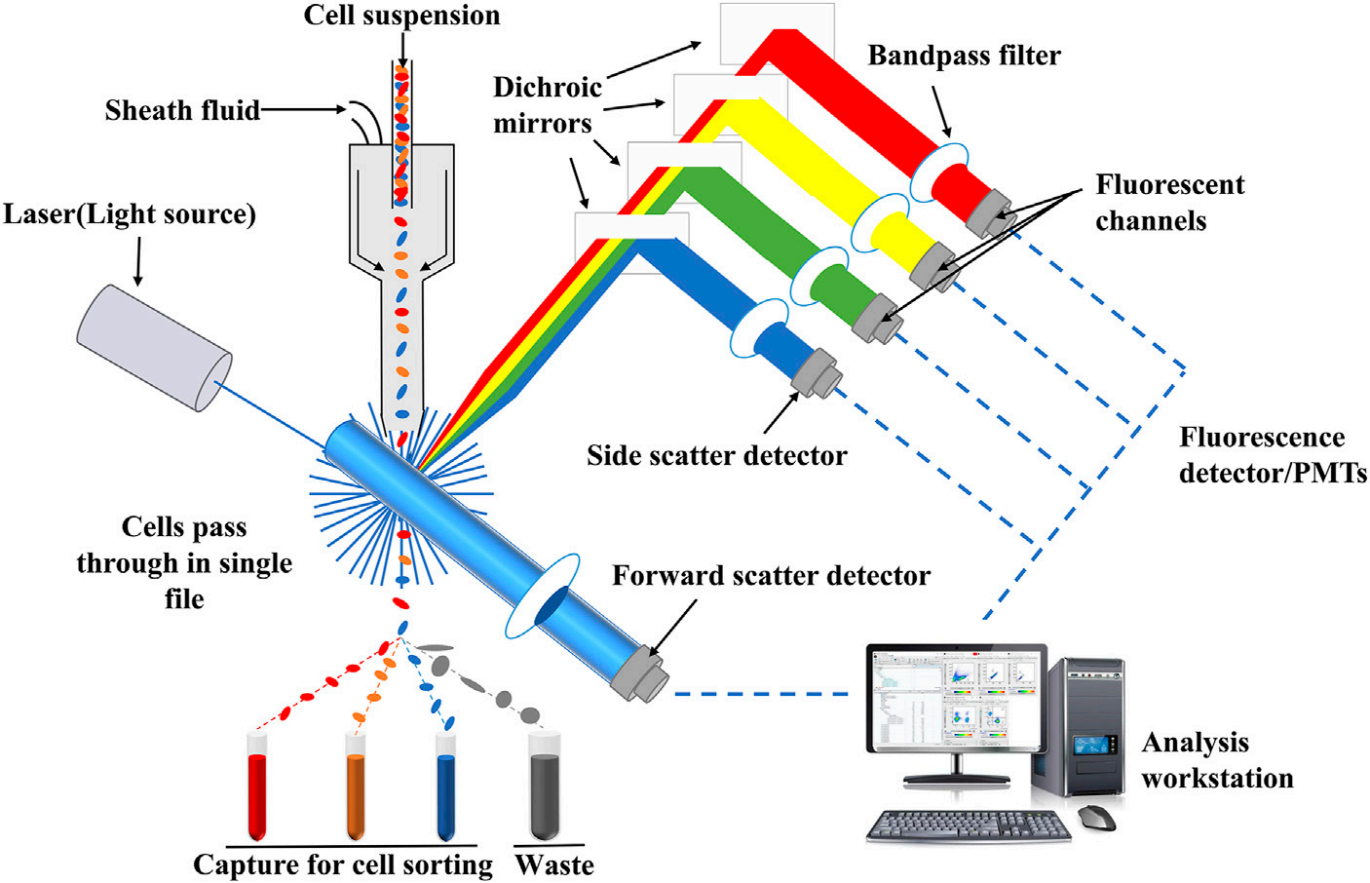
Schematic diagram of flow cytometry. Fluorescence signals which reflect the intensity of cell membrane surface antigen can be captured by fluorescence detector, and then, amplified and converted into an electrical signal by a photomultiplitizer. The continuous electrical signal is converted into a digital signal which can be recognized by a computer and processed into an image for in-depth analysis.

Flow cytometry analysis technology, one of the qualitative and quantitative analysis of advanced cell technology, is a detection method that can quickly determine the biological properties of cells or biological particle under a fast linear flow state (Ormerod, 2002). It has the advantages of high sensitivity, fast analysis, high accuracy, wide measurement parameters and high throughput (Adan, et al., 2017; McKinnon, 2018; Feng and Zhao, 2021). The principle of flow cytometry analysis technology is relatively simple, and its schematic diagram is shown in Figure 5. Flow cytometry is mainly composed of flow chamber, liquid flow system, light source, optical system, signal collection, signal conversion system, computer and analysis system (Jani et al., 2001). After suspension samples (body fluid, blood, etc.) or single cell stained with fluorescence dyes are prepared into suspension samples, they are wrapped in sheath liquid and passed through the detection area in a single row. Fluorescence signals, which reflect the intensity of cell membrane surface antigen, are captured by fluorescence detector, and then, amplified and converted into an electrical signal by a photomultiplitizer. The continuous electrical signal is converted into a digital signal which can be recorded by a computer and processed into an image for in-depth analysis (Yang et al., 2017; Shi M. et al., 2021).

Recently, with the development of science and technology, the performance of flow cytometer has been constantly improved, and the operation has become more and more flexible. It has become an indispensable detection tool in medical research and is often used in cancer treatment research in TCM. Fan et al. (Fan et al., 2020) found that dihydroartemisinin (DHA) could inhibit GC cell proliferation by flow cytometry combined with other technologies. DHA inhibited the occurrence and invasion of GC by regulating the cyclin D1-CDK4-Rb signaling pathway, which provided a guiding strategy for GC therapy. Meanwhile, Gong et al. (Gong et al., 2021) found that curcumin at the doses of 5, 15 and 30 μM could induce the apoptosis of renal carcinoma cells in a dose-dependent manner by flow cytometry. Zhang et al. (Zhang et al., 2017) investigated the effects of different concentrations of puerarin on the survival and apoptosis of SMMC-7721cells by combining MTT method with flow cytometry. The results showed that puerarin inhibited the proliferation of SMMC-7721 cells in a dose- and time-dependent manner, suggesting that puerarin may be a potential anti-hepatocellular carcinoma substance. Sun et al. (Sun and Liu, 2021) investigated the apoptosis of human tongue squamous cell within 24 h by flow cytometry, and found that osthole induced cell apoptosis in a concentration-dependent manner, which was attributed to the promotion of apoptosis, inhibition of autophagic flow and autophagy pathway injury. Zhu et al. (Zhu et al., 2021) investigated the role of trichosanthin (TCS) in cervical cancer by CCK-8 method and flow cytometry. They found that TCS inhibited cell viability, induced cell apoptosis as well as cell migration in a time- and dose-dependent manner. Similarly, Huang et al. (Huang et al., 1999) used flow cytometry to investigate the effect of Dongxia Pill on 116 tumor cell lines including nasopharyngeal carcinoma cells *in vitro*. They found that Dongxia Pill had significant inhibitory effect on the growth of 6 human tumor cells, and the inhibited rate increased with the increase of drug concentration. Moreover, Dongxia Pill could increase the G2 phase of nasopharyngeal carcinoma cells and induce cell apoptosis. Moreover, Hsu et al. (Hsu et al., 2008) used flow cytometry to investigate the anticancer effects of Huang-lian-jie-du-tang on HepG2 and PLC/PRF/5 cell. Huang-lian-jie-du-tang significantly reduced the levels of cyclin A, cyclin B1, Cdc2 and Cdc25C, resulting in cell cycle arrest. Meanwhile, the expression of Bcl-XL in HepG2 and PLC/PRF/5 cells were all decreased, thus inhibiting cell survival signal. It is suggested that Huang-lian-jie-du-tang has potential anticancer effect on human liver cancer cells.

Flow cytometry analysis technology had been extensively applied in TCM study due to its advantages of fast detection, high precision and accuracy (McKinnon, 2018). The target phenotype for tumor cell subsets (including lineage and functional balance) can be determined through multiple parameter flow of flow cytometry. Moreover, the effects of drugs on cytokines, cell adhesion molecules expression, immune related cell subsets, intracellular enzyme expression can be also investigated by flow cytometry. However, flow cytometry analysis technology requires samples to be in single-cell suspension, otherwise it will affect the experimental results (Otto et al., 2016; Ozawa et al., 2016). This will limit some of its application in TCM study such as *in vivo* evaluation.

### Other technologies

As mentioned above, nanotechnology, gene editing technology, RTCA technology, and flow cytometry analysis technology have all been extensively applied in TCM for cancer therapy, promoting their research process. Besides, the live cell dynamic imaging technology and cell energy metabolism technology have also been applied in TCM study.

The live cell dynamic imaging technology integrates the incubator and microscope system to keep cells viable, allowing long-term dynamic process experiments of cell migration, interaction, and response to environmental perturbations. At present, it is mainly used in the detection of dynamic processes of living cells such as cell proliferation/inhibition, cell migration/invasion, cell culture quality control/culture optimization, etc. (Li et al., 2021).

Energy metabolism has been recognized as a key factor in cellular function, from tumorigenesis to immune cell activation. Cell energy metabolism technology can simultaneously measure and analyze mitochondrial oxygen consumption rate (OCR) and extra cellular acidification rate (ECAR) in living cells in real-time, quantitatively and automatically without invading and destroying the sample (Yi et al., 2012; Pelletier et al., 2014). This technology is often used to study the dependence of cancer cells on energy-providing substrates (Patsoukis et al., 2015). The most common of which is the dependence of cancer cells on glutamine, which can reveal the weakness of cancer cells and thus provide a basis for finding drug targets (Hensley et al., 2013; Wang et al., 2015).

## Summary and perspective

In recent decades, increasing numbers of patients have been attracted to TCM for cancer treatment because TCM can regulate oncogenes and tumor suppressor genes, and microenvironment. Some current clinical trials of TCM in cancer treatment, were summarized in this review, as shown in Table 2 (The data were obtained from https://clinicaltrials.gov/). In order to promote the development of TCM in cancer therapy, some advanced technologies including nanotechnology, gene editing technology, RTCA technology, flow cytometry analysis technology had been widely applied in recent years. In this review, we summarize the application of these advanced technology in TCM for cancer therapy. Owing to poor solubility, low bioavailability, short half-life of some ingredient in TCM, nanotechnology was introduced to develop into various TCM-NDDS. The shortcomings of TCM can be effectively solved, and the medicinal value of TCM to treat and prevent cancer is enhanced. In addition, CRISPR/Cas9 technology, as an emerging technology, has also been extensively used in cancer research due to its advantages of simple design, high efficiency, low cost and simultaneous multi-point editing. CRISPR/Cas9 technology can achieve editing of any genomic site by synthesizing sgRNA that targets to various genomic sites (Concha-Benavente and Ferris, 2017; Shi S. J. et al., 2021), reducing the difficulties of tumor therapy research. Moreover, the roles of proteins in apoptosis and necroptosis can also be investigated through CRISPR/Cas9 technology, which promote the mechanism research of cancer. Besides, as a new cell detection technology, RTCA technology can be used to monitor the biological state and changes of cells, and cell dynamics in real time without labeling and damaging. The function and growth rule of different cells can also be determined. And owing to high sensitivity, fast analysis, high accuracy and large throughput, flow cytometry analysis technology is used to qualitatively and quantitatively analyze of the biological properties of cells. These advanced technologies all promote the research process of TCM for cancer therapy. However, some challenges still exist, and many efforts still need to be performed. For instance, the encapsulation efficiency of some TCM-NDDS is low, and the targeting is lack. Therefore, the preparation methods of TCM-NDDS need to be further explored and their preparation parameters should be further optimized. In addition, CRISPR/Cas9 technology shows a high off-target efficiency, and the off-target site is also generally difficult to find. RTCA can only indirectly monitor the activity of adherent cells, and special microplates are required when the RTCA instruments was used in different experiments, therefore increasing its cost. In view of this situation, some advanced technologies still need to be developed and introduced to study TCM for the treatment of cancer. Meanwhile, a variety of technologies can be combined to investigate the mechanism of inhibiting tumor growth and inducing tumor cell apoptosis. Only integrating with modern science and technology, and making full use of RTCA, flow cytometry technology and other advanced technologies, can TCM accelerate the pace of modernization and internationalization.

**TABLE 2:** The current clinical trials of TCM in cancer treatment. The date are obtained from https://clinicaltrials.gov/.

TCM	Cancer	NCT number	Clinical phase
Placebo Formulation	Triple Negative Breast Cancer	NCT04403529	Phase 3
TCM	Breast Cancer	NCT04438564	Not Applicable
	Colorectal Cancer		
	Cancer of Ovary		
	Cancer of Endometrium		
Acupoint with TCM	Rectal Cancer	NCT04749381	Phase 2
Acupoint with placebo			
YiQiFang	Non Small Cell Lung Cancer	NCT02900742	Phase 3
YangYinFang			
YiQiYangYinFang			
Gemcitabine^®^			
Pemetrexed^®^			
Docetaxel^®^			
Placebo Granules			
Letrozole	Breast Cancer	NCT02455154	Phase 2
Zhongyaofufang			
Xianlinggubao			
JinFuKang	Non-Small Cell Lung Cancer	NCT02603003	Phase 1
Cisplatin			
Pemetrexed			
Herba Scutellaria Barbatae	Breast Cancer	NCT00028977	Phase 1
			Phase 2
Tonifying Spleen and Kidney Sequential Regimen	Colon Cancer	NCT03716518	Phase 3
Placebo of ‘Tonifying Spleen and Kidney Sequential Regimen			
Chinese Herbs	Breast Cancer	NCT02011880	—
Huaier Granule	Breast Cancer	NCT02627248	Phase 4
Epirubicin			
Docetaxel			
Cyclophosphamide			
